# Murine esBAF chromatin remodeling complex subunits BAF250a and Brg1 are necessary to maintain and reprogram pluripotency-specific replication timing of select replication domains

**DOI:** 10.1186/1756-8935-6-42

**Published:** 2013-12-13

**Authors:** Shin-ichiro Takebayashi, Ienglam Lei, Tyrone Ryba, Takayo Sasaki, Vishnu Dileep, Dana Battaglia, Xiaolin Gao, Peng Fang, Yong Fan, Miguel A Esteban, Jiong Tang, Gerald R Crabtree, Zhong Wang, David M Gilbert

**Affiliations:** 1 Department of Biological Science, Florida State University, 319 Stadium Drive, Tallahassee, FL, 32306, USA; 2 Cardiovascular Research Center, Massachusetts General Hospital, Harvard Medical School, Richard Simches Research Center, 185 Cambridge Street, Boston, MA, 02114, USA; 3 Harvard Stem Cell Institute, 1350 Massachusetts Avenue, Cambridge, MA, 02138, USA; 4 Department of Cardiac Surgery, Cardiovascular Research Center, University of Michigan Medical School, North Campus Research Complex, Ann Arbor, MI, 48109, USA; 5 Stem Cell and Cancer Biology Group, Key Laboratory of Regenerative Biology, South China Institute for Stem Cell Biology and Regenerative Medicine, Guangzhou Institutes of Biomedicine and Health, Chinese Academy of Sciences, Guangzhou, 510530, China; 6 Howard Hughes Medical Institute, Stanford University School of Medicine, Stanford, CA, 94305, USA

**Keywords:** Replication domains, Replication timing, esBAF complex, Chromosome, Developmental regulation

## Abstract

**Background:**

Cellular differentiation and reprogramming are accompanied by changes in replication timing and 3D organization of large-scale (400 to 800 Kb) chromosomal domains (‘replication domains’), but few gene products have been identified whose disruption affects these properties.

**Results:**

Here we show that deletion of esBAF chromatin-remodeling complex components BAF250a and Brg1, but not BAF53a, disrupts replication timing at specific replication domains. Also, *BAF250a*-deficient fibroblasts reprogrammed to a pluripotency-like state failed to reprogram replication timing in many of these same domains. About half of the replication domains affected by Brg1 loss were also affected by BAF250a loss, but a much larger set of domains was affected by BAF250a loss. esBAF binding in the affected replication domains was dependent upon BAF250a but, most affected domains did not contain genes whose transcription was affected by loss of esBAF.

**Conclusions:**

Loss of specific esBAF complex subunits alters replication timing of select replication domains in pluripotent cells.

## Background

Developmental changes in chromosome structure can occur at the level of large, often megabase-sized chromosome domains [1-5]. This cell type-specific chromosomal domain structure is thought to be important for coordinating expression of genes, thereby ensuring proper development of embryos. However, the mechanisms regulating large-scale changes in chromosome structure during development are poorly understood. In particular, very few gene products have been found to be necessary to maintain structure and function of chromosomes at this level of organization.

The temporal order of replication (replication timing) is linked to many basic cellular processes that are regulated both during the cell cycle and development. We have developed a simple and robust assay to measure replication timing genome-wide [6,7]. We found that 400 to 800 Kb-sized replication domains are spatio-temporally reorganized genome-wide during embryonic stem (ES) cell differentiation into various cell lineages [6,8]. Similar sized replication domains are also misregulated in leukemia [9]. Cell type specific reorganization of replication domains is generally coordinated with transcriptional changes and is conserved between mouse and human [10-12]. Replication domain reorganization is also observed during iPSC generation in which somatic cell specific replication domain structure is erased and ESC-specific replication domain structure is re-established [8]. Considering that replication domains are regulated in the context of development and disease, it is presumed that epigenetic mechanisms play an important role in the formation of replication domain structure. However, in mammals, little or no effect on replication timing regulation has been reported for many chromatin modifier mutants, while these mutations significantly affect gene expression patterns [13-15]. Recently the first gene products with widespread effects on global replication timing in yeast (Fkh1/2 and Rif1) and mammals (Rif1) were identified [16-19]. Other gene products have been shown to have small effects on pericentric heterochromatin replication (Sub39h1/2 and G9a) [13,14]. Finally, replication timing of rDNA was shown to be affected by mutations in the rDNA-specific chromatin remodeling complex NoRC [20]. Together, these results suggest that specific gene products should eventually be identified that regulate cell type and domain-specific affects. Inspired by the specific and dramatic effect of NoRC on regulation of rDNA replication timing, we investigated the role of cell type specific chromatin remodeling complexes in replication timing changes during embryonic stem cell differentiation.

Brahma-associated factor (BAF) complexes are members of SWI/SNF ATP-dependent chromatin-remodeling family and regulate access of transcription factors by modulating chromatin structure. Of particular interest is that BAF subunits undergo compositional and stoichiometric change during mammalian development, which confers unique and essential roles to the complexes in cell fate determination [21-24]. For example, BAF155, BAF250a, and Brg1 are highly expressed in ESCs and their expression decreases significantly when ESCs differentiate, suggesting that these components may be essential for keeping ESCs in the undifferentiated ‘ground state’ [25]. In fact, Brg1 and BAF155 significantly promote reprogramming of mouse embryonic fibroblasts (MEFs) in combination with Yamanaka factors (Oct4, Sox2, Klf4, and c-Myc) [26]. BAF components are also instrumental for tissue-specific differentiation. The proper switch of neuron-specific BAF53 and BAF45 isoforms determines either the self-renewal or differentiation of neuron progenitor cells [27] and can convert fibroblasts to neurons [28]. Ectopic expression of BAF60c, a cardiac-enriched subunit, along with transcription factors GATA4 and TBX5, can convert non-cardiogenic mesoderm into beating cardiomyocytes [29]. These studies suggest that tissue-specific BAF complexes create chromatin environments favorable for transcription factor access.

In this study, we found that the embryonic stem cell-specific BAF complex (esBAF) complex deficiency leads to alterations of replication timing both in ESCs and during cellular reprogramming. Loss of DNA binding of the complex, but not transcriptional changes, correlated with changes in replication timing. These findings demonstrate the importance of chromatin remodeling complexes for maintaining replication-timing programs and, by proxy, large-scale chromatin reorganization.

## Results and discussion

### BAF250a is required to maintain replication timing at specific domains in embryonic stem cells

We first examined the effect of acute BAF250a loss on replication timing. BAF250a is essential for early embryogenesis and has shown to be involved in the recruitment of esBAF to its target sites [30,31]. We generated a cell line in which both homologues of *BAF250a* undergo simultaneous conditional deletion. In these cell lines, exon 8 of the *BAF250a* gene is flanked by 2 loxp sites and Cre recombinase (Mer-Cre-Mer) is induced upon addition of 4-hydroxytamoxfen (OHT), resulting in frameshift mutation followed by non-sense mediated decay. BAF250a protein level was rapidly and homogeneously diminished within 24 h and was undetectable 72 h after OHT treatment [see Additional file 1A].

**Figure 1:**
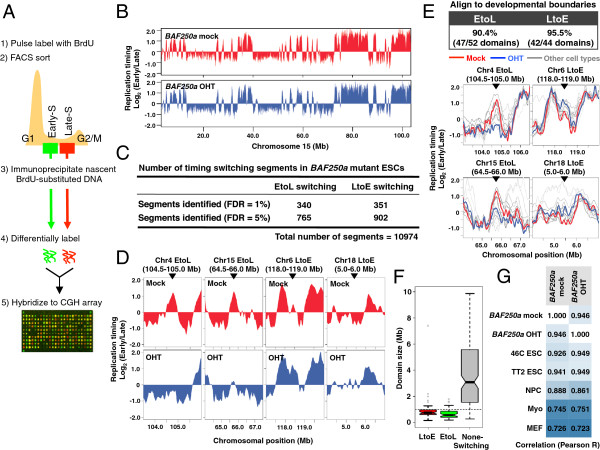
**Genome-wide replication timing analysis identifies a subset of BAF250a sensitive replication domains. (A)** Flow chart of genome-wide replication timing analysis [7]. BrdU-substituted DNA from early and late S phase cells was differentially labeled and hybridized to a whole-genome oligonucleotide microarray. **(B)** Replication timing profiles from mock- and 4-hydroxytamoxifen (OHT)-treated *BAF250a flox/flox* embryonic stem cells (ESCs) for chromosome 15. The signal ratio of early and late (Log2 early/late) for each probe is plotted against the chromosomal position. Shown are loess-smoothed plots for the average of two biological replicate experiments. **(C)** Summary of significant early to late (EtoL) and late to early (LtoE) switching segments in OHT-treated cells. Replication timing data were averaged into 200-Kb windows and statistical significance was calculated between mock and OHT-treated ESCs, as described in Methods. **(D)** Expanded plots for exemplary regions that undergo replication-timing switches in response to BAF250a loss are shown below. **(E)** BAF250a-dependent timing switching domains align to developmental boundaries. Replication timing plots of exemplary EtoL and LtoE switching domains shown in Figure 1D (mock-red and OHT-blue) are compared to replication timing profiles from other cell types (gray). The top table shows the percentage of EtoL and LtoE switching domains that align to developmental boundaries. **(F)** Box plots show the sizes of EtoL and LtoE timing switching domains compared to non-switching domains. **(G)** Correlation of replication timing datasets (Pearson’s R values).

Genome-wide replication timing analysis (Figure 1A, [7]) identified a set of genomic regions that changed replication timing either from early to late (EtoL) or from late to early (LtoE) in response to BAF250a loss after 72 h but not after 24 h (Figure 1B-D and [see Additional file 1B-C]). Observed changes in replication timing were highly reproducible between replicates [see Additional file 1D]. Since the changes were not as extensive as developmental changes [6,8], we calculated the *P* values for replication timing changes of 10,974 200-Kb segments, and applied a False Discovery Rate (FDR). Using this method, 691 and 1,667 200-Kb segments were identified as significantly changing replication timing at a 1% and 5% FDR, respectively (Figure 1C and [see Additional file 1E]). All affected segments examined aligned in register to domains of differential replication in one or more tissues during normal development (Figure 1E) and encompassed 400 to 800 Kb genomic segments (Figure 1F), consistent with domains whose replication timing is normally regulated during development [8-10]. We conclude that BAF250a is required to maintain normal developmental control of replication timing for a fraction of the ESC genome.

Several of the EtoL regions recapitulated developmental changes that occur during ES cell differentiation, raising the possibility that the observed changes might be an indirect result of cell differentiation after BAF250a loss. Indeed, it is known that BAF complex deficiency induces cell differentiation toward the primitive endoderm lineage after several rounds of cell division [30]. However, during the considerably shorter 72-h induction period, *BAF250a*-disrupted ESCs retained a higher genome-wide correlation in replication timing profile with pluripotent cell types than differentiated cell types (Figure 1G). For example, pluripotency-associated *Dppa2/4* and *Rex1* domains, which rapidly become late replicating during differentiation to every germ layer [8], retained ESC-specific early replication [see Additional file 2]. Moreover, we did not observe significant changes in the expression level of pluripotency-associated genes [see Additional file 3]. Together, we conclude that mutant ESCs still globally maintain an overall pluripotent cell replication timing program at least 72 h after OHT treatment, while specific domains require esBAF to maintain their replication time.

### BAF250a is required to re-establish replication timing of an overlapping set of select domains during somatic cell reprogramming

**Figure 2:**
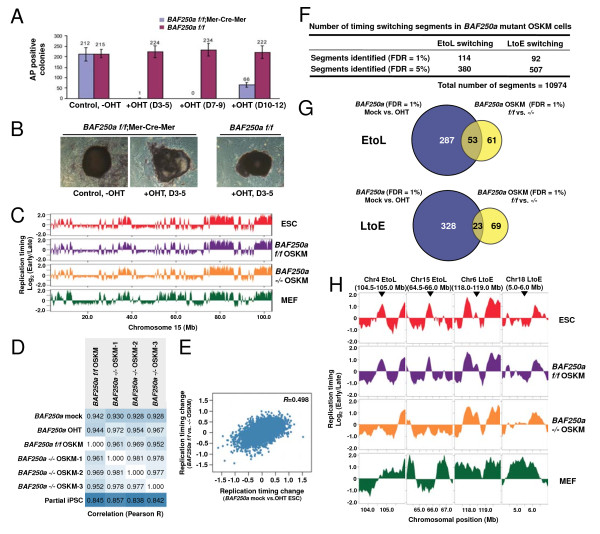
**Role of BAF250a in the regulation of replication domains in cells undergoing somatic cell reprogramming. (A)** Number of alkaline phosphatase (AP)-positive colonies derived from 4-hydroxytamoxifen (OHT)-treated *BAF250a flox/flox* and *BAF250a flox/flox*; *Mer-Cre-Mer* mouse embryonic fibroblasts (MEFs) after Oct4, Sox2, Klf4, and c-Myc (OSKM) expression. **(B)** Morphology of AP-positive colonies derived from *BAF250a flox/flox* and *BAF250a flox/flox*; *Mer-Cre-Mer* MEFs. **(C)** Replication timing profiles of control embryonic stem cells (ESCs) (mock-treated ESCs from Figure 1), *BAF250a flox/flox* OSKM, *BAF250a -/-* OSKM, and MEFs. Replication timing was analyzed in two independent *BAF250a flox/flox* OSKM clones and three independent *BAF250a -/-* OSKM clones, and the averaged data are shown for each genotype. **(D)** Correlation analysis between replication timing datasets (Pearson’s R values). **(E)** Replication timing changes of BAF250a-affected domains in OSKM cells (*BAF250a -/-* ratio - *BAF250a flox/flox* ratio) were plotted against the same changes in ESCs (OHT ratio - mock ratio). **(F)** Summary of significant early to late (EtoL) and late to early (LtoE) switching segments in OHT-treated cells determined as in Figure 1C.** (G)** Venn diagram showing the overlap between genomic segments that undergoes EtoL switching (top) and LtoE switching (bottom) upon BAF250a loss in ESCs versus OSKM cells (FDR = 1% segments from Figure 1C and Figure 2F). **(H)** Replication timing plots of exemplary EtoL and LtoE switching domains.

To further confirm the requirement of BAF250a for replication timing of specific domains in pluripotent cells, we investigated whether similar replication timing defects occur in cells reprogrammed from somatic cells in the absence of BAF250a. First we examined the effect of BAF250a loss on formation of iPSC-like colonies. *BAF250a flox/flox* and *BAF250a flox/flox Mer-Cre-Mer* MEFs were treated with OHT at Days 3 to 5 after virus-mediated transduction of reprogramming factors (Oct4, Sox2, Klf4, and c-Myc; OSKM). We observed a significant decrease in the number of alkaline phosphatase (AP)-positive colonies derived from cells expressing Mer-Cre-Mer compared to control cells (Figure 2A and B), demonstrating that BAF250a is required for efficient somatic cell reprogramming, or for growth and viability of the latter. Also, colonies from OHT-treated *BAF250a flox/flox Mer-Cre-Mer* cells have an irregular unsmoothed shape (Figure 2B), which is reminiscent of the morphology of *BAF250a*-deficient ESCs [30]. Several iPSC-like colonies from *BAF250a flox/flox* and *BAF250a flox/flox Mer-Cre-Mer* cells were genotyped and confirmed to be *BAF250a flox/flox* and *BAF250a -/-*, respectively. These colonies, which we refer to as *BAF250a flox/flox* OSKM and *BAF250a -/-* OSKM, were also confirmed to express high levels of ESC pluripotency markers (data not shown).

Next we performed replication-timing analysis of *BAF250a flox/flox* OSKM and *BAF250a -/-* OSKM cells. Despite the fact that loss of BAF250a significantly reduced the efficiency of AP-positive colony production, the genome-wide replication timing profiles of three independent AP-positive *BAF250a* -/- OSKM clones were almost identical to that of control *BAF250a flox/flox* OSKM or other ESC lines and were clearly more similar to pluripotent cells than to partially reprogrammed iPSCs (piPSCs; Figure 2C and D). This result suggests that *BAF250a -/-* OSKM have passed the common epigenetic block experienced by piPSCs [8]. Nonetheless, *BAF250a -/-* OSKM cells display distinct replication timing differences from ESCs or control OSKM cells (Figure 2E-H). When replication timing differences in OSKM cells are compared to those in ESCs, we observed a conservation of BAF250a-affected domains between ESCs and OSKM cells (Figure 2E). Indeed, we identified a set of chromosomal domains that undergo replication timing switching in *BAF250a*-deficient OSKM cells (Figure 2F) and found that significant fraction of these switching domains overlap with those identified in *BAF250a*-deficient ESCs (Figure 2G and H). These results confirm a role for BAF250a in replication timing regulation of specific chromosomal domains in the pluripotent state.

### Loss of Brg1, but not BAF53a, affects an overlapping set of replication domains

**Figure 3:**
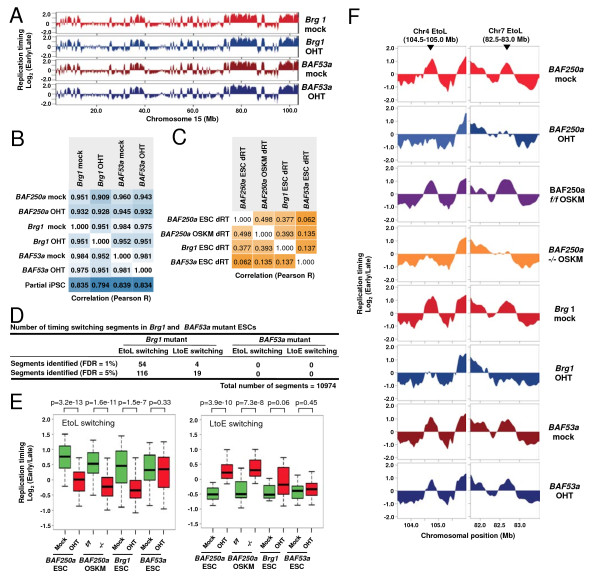
**Brg1 but not BAF53a is required for the regulation of replication domains. (A)** Replication timing profiles of mock- and 4-hydroxytamoxifen (OHT)-treated *Brg1 flox/flox* and *BAF53a flox/-*, displayed as in Figure 1B. Plots for the average of two biological replicate experiments are shown. **(B)** Correlation analysis between replication timing datasets (Pearson’s R values). **(C)** Correlation analysis of replication timing differences (dRT). Replication timing data were averaged into 200-Kb windows and dRTs (that is, *Brg1* OHT ratio - *Brg1* mock ratio) were calculated for comparisons between different groups. A substantially lower conservation of replication timing affected regions is seen between *BAF53a* ESCs and *BAF250a* or *Brg1* embryonic stem cells (ESCs) than between *BAF250a* and *Brg1* ESCs. **(D)** Summary of significant early to late (EtoL) and late to early (LtoE) switching segments in OHT-treated cells as determined in Figure 1C.** (E)** Box plots show the replication timing of segments that are sensitive to BAF250a loss (false discovery rate (FDR) = 1% domains identified in Figure 2G) in various esBAF subunit mutants. Replication timing in mutants (red) is compared to that in control counterparts (green). *P* values were calculated using the Wilcoxon rank sum test. **(F)** Replication timing plots of exemplary EtoL switching domains. These domains undergo EtoL switching in *BAF250a* and *Brg1* mutants, but not in *BAF53a* mutant.

Since BAF250a is a subunit of the esBAF complex in ESCs [32], we next examined the role of two other esBAF subunits, Brg1 (catalytic ATPase subunit) and BAF53a (another ESC-specific subunit), in the regulation of replication domain structures. To this end, we performed genome-wide replication timing analysis in *Brg1* and *BAF53a* conditional knockout ESC lines [33,34]. Similar to *BAF250a* knockouts, the overall replication timing profiles of OHT-treated *Brg1 flox/flox* and *BAF53a flox/-* ESCs are almost identical to that of parental (mock-treated) ESCs, suggesting an overall maintenance of stem-cell identity in these cells (Figure 3A and B). This was further confirmed by the finding that expression of Oct4 did not change in these mutant cells [see Additional file 4]. However, we found that loss of Brg1 induced altered replication timing profiles in a subset of chromosomal domains with a bias for EtoL switching (Figure 3C and D) and that these Brg1-sensitive domains significantly overlap with BAF250a-sensitive domains (Figure 3C and [see Additional file 5]). On the other hand, loss of BAF53a did not induce any significant changes in replication timing (Figure 3C and D). This latter result, coupled with our previously published results with other Cre-inducible deletions [15], confirms that the changes in replication timing are not due to the activation of Cre recombinase by OHT treatment.

The fraction of chromosome domains that displayed EtoL switching in response to BAF250a loss (commonly misregulated in *BAF250a*-deficient ESCs and OSKM cells), showed a very similar tendency of replication timing switching in *Brg1* but not *BAF53a* mutant ESCs (Figure 3E-F). For example, at chromosome 4 (104.5-105.0 Mb) and chromosome 7 (82.5-83.0 Mb) domains where the BAF250a is required for early replication in both ESCs and OSKM cells, these domains are late replicating after Brg1 loss, while they remain early replicating in the absence of BAF53a (Figure 3F). Together, these results demonstrate a BAF53a-independent function of the esBAF complex is required for proper regulation of replication timing at specific replication domains. However, the partial overlap in affected regions between Brg1 and BAF250a suggests the potential for independent roles of each subunit or gain of function effects of each subunit in the absence of the other.

### BAF250a-dependent binding of esBAF complexes to affected domains independent of transcriptional regulation

**Figure 4:**
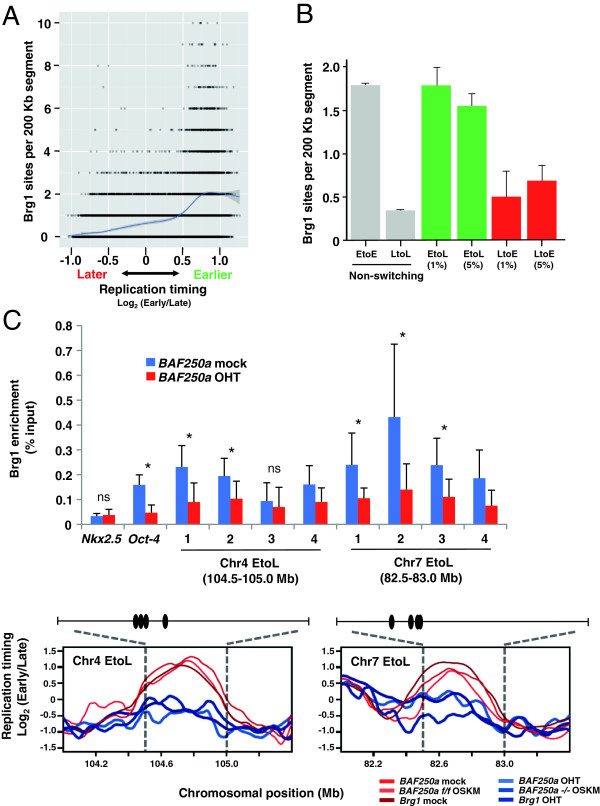
**BAF250a-dependent Brg1 enrichment in early to late (EtoL) switching domains. (A)** Genome-wide relationship between Brg1 enrichment and replication timing. The x-axis shows the embryonic stem cell (ESC) replication timing data averaged in 200-Kb windows and the y-axis shows the number of Brg1 binding sites in each corresponding window. The smoothed blue line in the plot shows the average number of Brg1 sites per 200-Kb segment (thicker gray line shows the standard error). **(B)** Average number of Brg1 binding sites per 200-Kb segment that switched EtoL or LtoE after loss of Brg1 (FDR = 1% and 5% segments from Figure 3D) and comparison to the remainder of the genome (‘non-switching’). Based on this data, 400 to 800 Kb EtoL switching domains are thought to have 3.5 to 7.0 Brg1 binding sites. **(C)** BAF250a-dependent Brg1 enrichment within the EtoL domain (chr4: 104.5 to 105.0 Mb and chr7: 82.5 to 83.0 Mb from Figure 3F), revealed by Brg1 chromatin immunoprecipitation (Brg1-ChIP). Brg1 enrichment was analyzed at multiple sites within the chr4 domain (site-1: 104654835 to 104654965, site-2: 104668986 to 104669071, site-3: 104693231 to 104693309, site-4: 104713676 to 104713776) and chr7 domain (site-1: 82610306 to 82610419, site-2: 82647473 to 82647844, site-3: 82660145 to 82660243, site-4: 82662755 to 82662863) both in mock-treated (blue) and 4-hydroxytamoxifen (OHT)-treated (red) *BAF250a flox/flox* ESCs. These are Brg1 binding sites identified by ChIP-seq [35] and their positions relative to EtoL domains are shown under the Brg1-ChIP result. The binding of Brg1 at *Oct4* and *Nkx2.5* promoters was used as positive and negative controls, respectively. The *Oct4* promoter region also showed a BAF250a-dependent Brg1 binding. *P <0.05; ^ns^*P* >0.05 (no significant difference). Statistical analysis was performed by a two-tailed Student’s t-test.

Since disruption of BAF complexes have a profound effect on genome-wide transcriptional regulation in ESCs [30,33,35], we wished to determine whether the effect of BAF250a loss on replication timing was linked to altered gene expression in these domains. Brg1 ChIP-seq data in mESCs [35] revealed significant enrichment of Brg1 proteins in early replicating regions of the ESC genome (Figure 4A). We observed significant enrichment of Brg1 in Brg1-sensitive EtoL domains compared to LtoE domains, though this enrichment level is comparable to that seen in unaffected early replicating (EtoE) domains (Figure 4B). Unfortunately, several anti-BAF250a antibodies were not of sufficient quality for chromatin immunoprecipitation (ChIP). However, Brg1 ChIP at two of the BAF250a-sensitive early replicating domains, 500-Kb genomic segments on chromosome 4 (104.5-105.0 Mb) and chromosome 7 (82.5-83.0 Mb), revealed BAF250a-dependent Brg1 enrichment at multiple sites within these domains (Figure 4C). It should be noted that loss of BAF250a also impaired Brg1 enrichment at the Oct4 gene region that retains its time of replication (EtoE) regardless of BAF250a availability (Figure 4C and [see Additional file 6]). Thus, esBAF is generally enriched in early replicating domains, although loss of esBAF is not sufficient to elicit a change in replication timing at all domains.

**Figure 5:**
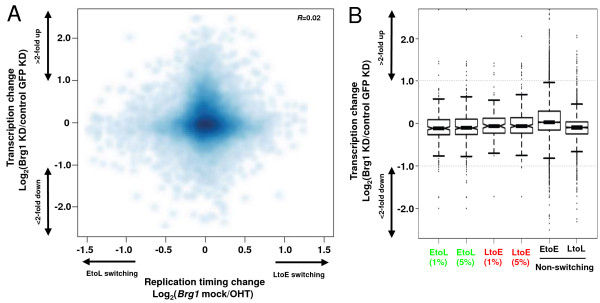
**Replication timing changes are not associated with transcriptional changes. (A)** Replication timing changes of Brg1-affected domains in ESC (4-hydroxytamoxifen (OHT) ratio - mock ratio) were plotted against transcriptional changes after Brg1 knockdown in embryonic stem cell (ESC) (Brg1 knockdown (Brg1 KD) ratio - control green fluorescent protein knockdown (GFP KD)) [35]. **(B)** Box plots show the transcriptional changes in Brg1-affected EtoL and LtoE domains (false discovery rate (FDR) = 1% and 5% segments from Figure 3D) compared to non-switching late (LtoL) or early (EtoE) domains.

Finally, we examined the effect of esBAF disruption on transcription of genes within the domains affected for replication timing [35]. None of the esBAF target genes in switching regions (0/19) was more than two-fold up- or downregulated upon Brg1 knockdown (Brg1 KD). Genes in switching regions, regardless of whether they were targets of esBAF binding, have no coordination between EtoL/LtoE changes and transcriptional downregulation/upregulation, respectively (overall R = 0.02) (Figure 5A and B). This is consistent with the hypothesis that replication timing is associated more with transcriptional competence than transcription per se [36] and suggests that the role of esBAF in regulating replication timing is not a direct transcriptional role for this complex. Taken together, these results demonstrate that BAF250a-dependent Brg1-containing esBAF complexes are recruited to regions that require BAF250a and Brg1 for early replication in ESCs, but most of these regions do not contain esBAF-regulated genes.

## Conclusions

In summary, our data presented here reveal an unanticipated effect of esBAF complex disruption on replication timing and, by proxy, higher-order chromatin folding [10,37,38]. Yeast transcription factors Fkh1 and Fkh2 are thought to modulate replication timing by bringing early replication origins in close proximity in the nuclear space independent of their transcriptional activity [16]. It is possible that the BAF complexes play a similar role in mammalian cells, thereby promoting the formation of an early replication domain. Indeed it has been shown that Brg1 is involved in cell type-specific chromatin loop formation at the *beta-globin* locus [39]. Interestingly, esBAF complexes are known to interact with the nuclear matrix protein Rif1 which has recently been identified as global replication timing regulators [18,19,32]. Currently, it is unclear why only a small subset of esBAF-enriched replication domains is sensitive to esBAF complex deficiency. For example, other early replicating domains harboring genes such as *Oct4* have multiple Brg1 binding sites but maintain their early replication in the absence of BAF250a or Brg1 [see Additional file 6]. This suggests that there are additional mechanisms maintaining early replication of these domains, whereas we have identified a subset of domains at which esBAF presence has a major effect on replication timing. This may be related to whether or not the affected domains are capable of switching replication timing, as none of the affected domains were constitutively early replicating (Figure 1E). Future studies are warranted to uncover the mechanism by which BAF complexes influence replication timing during stem cell self-renewal and differentiation.

## Methods

### Embryonic stem cell culture

*BAF250a flox/flox; Mer-Cre-Mer* ESC lines were established from day 3.5 blastocysts obtained by crossing *BAF250a flox/+; Mer-Cre-Mer* with *BAF250a flox/flox* and maintained on feeder MEFs in the presence of leukemia inhibitory factor (LIF) as described previously [30]. *Mer-Cre-Mer* mice were purchased from the Jackson Laboratory; Bar Harbor, ME USA (stock number: 008463). *Brg1 flox/flox; Actin-CreER* and *BAF53a flox/-; Actin-CreER* ESC lines were maintained as described previously [33,34]. To generate mutant ESCs, these ESC lines were treated with 1 μM 4-hydroxytamoxifen (OHT) for 24 h and harvested 48 h later, unless otherwise indicated. As a control, cells were treated with ethanol.

### Somatic cell reprogramming

MEF cells derived from *BAF250a flox/flox; Mer-Cre-Mer* and *BAF250a +/+; Mer-Cre-Mer* were infected with four reprogramming factors (Oct4, Sox2, Klf4, and c-Myc, OSKM) [40]. Early passage fibroblasts (less than passage 5) were cultured in 6-well dishes and about 4 × 10^4^ cells in each well were infected overnight with viral supernatants freshly prepared by transfection of the retroviral packaging Plat-E cell line (Lipofectaine 2000, Invitrogen, Life Technologies, Carlsbad, CA, USA) containing the cDNAs of the mouse reprogramming factors. Three days after infection, cells were passaged into new wells and tamoxifen was added for three days (Days 3 to 5) or other time windows to ablate BAF250a. Control iPSC-like colonies (*BAF250a +/+*, OHT treatment or *BAF250a flox/flox; Mer-Cre-Mer*, no OHT treatment) were typically picked 21 days after infection and iPSC-like colonies from *BAF250a flox/flox; Mer-Cre-Mer*, OHT treated fibroblast culture were typically picked 30 days post infection. Genotyping of *BAF250a* was performed by PCR. We used the primer sequences 5′-GTAATGGGAAAGCGACTACTGGAG-3′ and 5′-TGTTCATTTTTGTGGCGGGAG-3′, which amplify a 632-bp fragment from the WT locus, an 812-bp fragment from the floxed locus and a 298-bp fragment from the knockout locus, respectively. PCR reactions were carried out with 40 cycles (30 sec at 94°C, 30 sec at 59°C, 1 min at 72°C). For alkaline phosphatase (AP) staining, culture wells containing iPSC-like colonies were washed with PBS and cells were fixed with 4% paraformaldehyde in PBS for 2 min at 20°C. Fixed cells were then rinsed twice with 0.5 ml of TBST (TBS plus 0.05% Tween-20) and incubated with fresh AP staining solution (4.5 μl 50 mg/ml nitro blue tetrazolium, 3.5 μl 50 mg/ml 5-bromo-4-chloro-3-indolyl phosphate in 100 mM Tris–HCl, pH 9.5, 100 mM NaCl, 50 mM MgCl_2_) in the dark room at 25°C for about 15 min. Stained cells were rinsed with PBS and kept at 4°C.

### Chromatin Immunoprecipitation

ChIP was performed as previously described [41]. Two million cells were harvested and fixed in 1% formaldehyde for 10 min at 25°C, then stop fixation in 0.125 M glycine. Fixed cells were sonicated to produce chromatin fragments 300 to 700 bp in length. Chromatin fragments were then immunoprecipitated with anti-Brg1 antibody [42]. The precipitated DNAs were then purified by ethanol precipitation after phenol-chloroform extraction. Quantitative PCR reactions were performed to detect the occupancy of Brg1 at multiple sites within the chromosome 4 and 7 EtoL domains. Quantitative PCR reactions included the following: 4 μl of ChIP product (200 μl per ChIP assay), 10 μl of 2X SYBR green PCR master mix (Applied Biosystem, Carlsbad, CA, USA, 4309155) and 25 nM of each primer. QPCR reactions were tripled and performed in ABI StepOnePlus system through 50 cycles (15 sec at 95°C, 45 sec at 60°C). Ct values were generated by ABI software. Standard errors in Figure 4C were generated from six individual ChIP-qPCR experiments. Concentration of the ChIP samples was calculated as percent of input. QPCR was performed using primers for *Oct4* promoter (forward, 5′-AGTGAGAAGGGCAGGAGGAT-3′; reverse, 5′-CCTACTTGCTCACACCACCA-3′), *Nkx2.5* promoter (forward, 5′-CCACCCCCAACCCTGCGTTT-3′; reverse, 5′-AGGGGCCGCGACACATTTGG-3′), Chr4 site-1: 104,654,835-104,654,965 (forward, 5′- CAACAACCAACCTAGCTTTCCT-3′; reverse, 5′-GAGAGGATCGGTGGGAGGTC-3′), Chr4 site-2: 104,668,986-104,669,071 (forward, 5′- TCTGAGGGGGTTGGCATAGA-3′; reverse, 5′-GATGTGTGCAAATGGGACCG-3′), Chr4 site-3: 104,693,231-104,693,309 (forward, 5′-TCCCTTACGTCACCGTCTGA-3′; reverse, 5′-AAACACCTTGACCAGAGGGC-3′), Chr 4 site-4: 104,713,676-104,713,776 (forward, 5′-GTTGGCGCTTGTGAACTGAG-3′; reverse, 5′-GTTAGGCAATGGCAGGAGGT-3′), Chr7 site-1: 82,610,306-82,610,419 (forward, 5′-TCCTCGGGAACCTACTCCAG-3′; reverse, 5′-TACAGACACCGACTGAGGCT-3′), Chr7 site-2: 82,647,473-82,647,844 (forward, 5′-GCTCGGGTCTCTGTGTCTGTC-3′; reverse, 5′-CGGGTGGGAGAAAGTGGAAGA-3′), Chr7 site-3: 82,660,145-82,660,243 (forward, 5′-CTCTGCAGCCTGTAAGTGGT-3′; reverse, 5′-ATGTACCACCAGCACACCAG-3′), and Chr7 site-4: 82,662,755-82,662,863 (forward, 5′-CTGATGCCCTGTAGTGCCTT-3′; reverse, 5′-TACAGGGTGGAGGTGGCTTT-3′).

### Immunostaining

ES cells grown on culture dishes were collected by trypsinization, cytospun onto glass slides, fixed with 4% paraformaldehyde in PBS (10 min, 25°C), washed, and then permeabilized with 0.5% Triton X-100 in PBS (10 min, 25°C). For immunostaining, the samples were incubated in blocking solution (3% BSA, 0.1% Tween 20, 4 × SSC) for 30 min at 37°C to reduce nonspecific binding, and then in detection solution containing primary antibodies (1% BSA, 0.1% Tween 20, 4 × SSC) for 1 h at 37°C. After three washes with 4 × SSC, the samples were incubated in detection solution containing secondary antibodies. For Nanog immunostaining, cells were fixed with formalin/acetic acid and then treated with methanol for 20 min at -20°C. The primary antibodies were: anti-BAF250a mouse monoclonal antibody (Santa Cruz Biotechnology, Santa Cruz, CA, USA, sc-20701) diluted 1:50, anti-Oct4 mouse monoclonal antibody (BD Biosciences, San Jose, CA, USA, 611202) diluted 1:200, anti-Nanog rabbit polyclonal antibody (Chemicon, Temecula, CA, USA, MAB3448) diluted 1:20. Alexa Fluor 488 goat anti-mouse IgG (Molecular Probes, Life Technologies, Carlsbad, CA, USA, A11017) and Alexa Fluor 555 goat anti-rabbit IgG (Molecular Probes, Life Technologies, Carlsbad, CA, USA, A21430) were the secondary antibodies. Before imaging, the slides were counterstained with DAPI (200 ng/ml), washed with 4X SSC, and then mounted in 90% glycerol containing antifade reagent.

### RNA FISH

RNA FISH was performed as described previously [43]. To generate RNA FISH probes, *Rex1* genomic DNA fragments were amplified, cloned into pBluscript, and labeled by nick translation. Cells were treated with 0.5% Triton X-100 in CSK buffer (100 mM NaCl, 300 mM sucrose, 10 mM Pipes, pH 6.8, 3 mM MgCl_2_, 1 mM EGTA) for 30 sec at 4°C, fixed with 4% paraformaldehyde, and then immersed in 70% ethanol for 5 min at -20°C, dehydrated through a 90% and 100% ethanol series, and the denatured FISH probe mixture was hybridized to slides at 37°C for 16 h in a moist chamber. Slides were washed three times with 50% formamide in 2X SSC at 43°C and three times with 0.8X SSC at 60°C. Slides were then incubated for 30 min in a blocking solution (3% BSA, 0.1% Tween 20 in 2X SSC) at 37°C and incubated in a detection solution (in 1% BSA, 0.1%Tween 20 in 2X SSC) containing anti-digoxigenin-conjugated rhodamine (Roche, Nutley, New Jersey, USA, 11207750910) for 30 min at 37°C. Then slides were washed three times with 4X SSC, 0.1% Tween 20 for 5 min at 43°C. Before imaging, the slides were counterstained with DAPI (200 ng/ml), washed with 4X SSC, and then mounted in 90% glycerol containing antifade reagent.

### Replication timing profiling by microarray

Replication timing analysis was performed as described previously [6,7]. In brief, cells were labeled with 50 μM BrdU for 2 h, washed twice with ice-cold PBS, trypsinized, and then were fixed in 75% ethanol. These cells were resuspended in PBS containing 1% FBS, stained with propidium iodide (50 μg/ml) for 30 min in the presence of RNaseA (0.5 mg/ml), and then were sorted into early and late S phase fractions by flow cytometry. After phenol-chloroform extraction of DNA, immunoprecipitation with anti-BrdU mouse monoclonal antibody (BD Biosciences, San Jose, CA, USA, 555627) was performed in each fraction to enrich BrdU-substituted replicating DNA. Isolated early and late replicating DNA were amplified by whole-genome amplification (WGA) (Sigma-Aldrich, St Louis, MO, USA, GenomePlex), labeled with Cy3 and Cy5, and hybridized to a mouse whole-genome microarray (NimbleGen Symtems, Madison WIS, USA, 2006-07-26_MM8_WG_CGH or 100718_MM9_WG_CGH_HX3). Sample labeling, hybridization and data extraction were performed according to standard NimbleGen Systems procedures. Data analyses were performed using R/Bioconductor (http://www.r-project.org; http://www.bioconductor.org). Obtained raw datasets were normalized using the limma package in R/Bioconductor and loess-smoothed over a 300-Kb window size. These smoothed datasets were used to generate replication-timing plots in figures. For some analyses, datasets were averaged into 200-Kb windows (fixed position) and replication timing differential (that is, OHT ratio - mock ratio) was determined for each 200-Kb segment. In order to determine the significant replication timing switching domains that are independent of changes between replicates, we determined Euclidian distance at 10,974 200-Kb segments between groups (that is, mock versus OHT) and within groups (that is, mock replicate-1 versus mock replicate-2), which was used to calculate *P* values at each 200-Kb genomic segment. Statistical significance was then calculated using the qvalue package in R/Bioconductor, which yields a q-value for each segment that reflects the proportion of false-positives (False Discovery Rate; FDR) among segments deemed to have significant replication timing (RT) changes. High confidence replication timing switching domains were selected with a q-value cutoff of 0.01, corresponding to an overall FDR of 1%. A q-value cutoff of 0.05 was also used to identify a set of lower confidence domains. To examine alignment of timing switching domains to developmental domains, replication timing data from 9 cell types (ESC/iPSC, EBM3/EPL, EBM6/EpiSC, NPC, Mesoderm, Endoderm, partial iPSC, MEF, and Myoblast) were assembled from the ReplicationDomain.org database [44] and plotted together with the data from *BAF250a* mock and OHT. Timing switching domains from chromosome 1 (largest-sized) and chromosome 10 (middle-sized) were selected and their alignment to developmental domains was judged by visual inspection in Figure 1E. Indeed, when we examined statistical significance of replication timing changes of *BAF250a* OHT compared to other cell lines, most domains examined in Figure 1E were not significantly different from at least one of nine cell types, even with a q-value cutoff of 0.2 (42/52 EtoL domains and 42/44 LtoE domains). The size of switching domains was determined using a segmentation algorithm in the DNAcopy package in R/Bioconductor as described previously [6]. Unsmoothed datasets consisting of replication timing (*BAF250a* OHT ratio - mock ratio) for all probes were processed for switching domain segmentation and the resultant EtoL and LtoE segment sizes were shown in Figure 1F. Replication timing datasets are downloadable from ReplicationDomain (http://www.replicationdomain.org).

### Imaging system and measurement

Images were collected using a Nikon Ti-U Eclipse fluorescence microscope equipped with a 60x, 1.40 NA lens and a cooled charge-coupled device camera (C4742-95-12ER, Hamamatsu Photonics, Hamamatsu, Japan), controlled by a windows computer running the software program MetaMorph (Molecular Devices, Sunnyvale CA, USA).

## Abbreviations

BAF: Brg1/Brm associated factors; Brg1: Brahma-related gene 1; ChIP: Chromatin immunoprecipitation; ES cell: Embryonic stem cell; EtoL: Early to late; dRT: Replication timing differences; FDR: False discovery rate; GFP: Green fluorescent protein; iPSC: Induced pluripotent stem cells; KD: Knockdown; LtoE: Late to early; OHT: 4-hydroxytamoxifen; MEFs: Mouse embryonic fibroblasts; OSKM: Oct4, Sox2, Klf4, and c-Myc; RT: Replication timing

## Competing interests

The authors declare that they have no competing interests.

## Authors’ contributions

ST and DMG designed the research; ST, TS, DB carried out the replication timing experiments; ST, TR, and VD analyzed the replication timing data; IL carried out the Brg1 ChIP experiment; XG, PF, YF, MAE, JT, GRC, and ZW generated mutant cell lines; and ST and DMG wrote the paper. All authors read and approved the final manuscript.

## Supplementary Material

Additional file 1 **Replication timing profile at chr15 (65.4-66.0 Mb) domain. (A)** TOP: The BAF250a protein level was monitored by immunofluorescence staining at 0 (control), 24, and 72 h after 4-hydroxytamoxifen (OHT)-mediated induction of Cre recombinase. Circled are the nuclei of feeder mouse embryonic fibroblasts (MEFs), in which BAF250a protein level is not affected by the drug treatment, which serves as an internal immunostaining control. Bars, 10 μm. BOTTOM: Western blot showing protein levels of BAF250a with (OHT) and without (Mock) Cre recombinase induction. **(B)** Replication timing profile of the chr15 domain shown in Figure 1D from untreated, 24 h mock-treated and 24 h OHT-treated embryonic stem cells (ESCs). Replication timing change at this domain was not observed during the 24 h experimental period. (C) Box plots show the replication timing of domains that are sensitive to BAF250a loss (false discovery rate (FDR) = 1% from Figure 1C) after 24 h and 72 h of OHT treatment. **(D)** Responses to BAF250a loss are reproducible. Top panels show average replication timing profile at the Chr15 domain. Replication timing profiles from two independent experiments are shown below. **(E)***P* value calculation based on the global Euclidian distances between groups and within replicates. Top plot is an examplary region showing replication timing of *BAF250* ESC OHT in dark and light blue, and *BAF250* ESC mock in dark and light grey. Bottom plot shows global Probability Density Function (PDF) of Euclidian distance between groups (red) and within replicates (grey) calculated from replication timing in individual probes. The individual probes with significant replication timing differences are shown as red lines in the top plot. Click here for file

Additional file 2 ***BAF250a*-deficient embryonic stem cells (ESCs) have pluripotency-specific replication profiles.** Replication timing profile of *Dppa2/4* and *Rex1* domains derived from genome-wide analysis of various cell types. These domains are known to show early replication in pluripotent cells, but switch to late replication after differentiation [6]. Click here for file

Additional file 3 **Pluripotency-associated marker expressions in *BAF250a*-deficient embryonic stem cells (ESCs). (A)** Immunofluorescence analysis of Oct4 and Nanog proteins (left two panels) and RNA-FISH analysis of *Rex1* mRNA in mock- and 4-hydroxytamoxifen (OHT)-treated ESCs. Bars, 10 μm. **(B)** Western blot showing protein levels of Oct4. The results for the loading control, tubulin, were the same as those in Additional file 1A. **(C)** RT-PCR expression level validation for pluripotency-associated genes. *P <0.05; ^ns^*P* >0.05 (no significant difference). Statistical analysis was performed by a two-tailed Student’s t-test. Click here for file

Additional file 4 **Characterization of conditional *Brg1 *** **and *BAF53a *** **knockout.** Western blot showing protein levels of Brg1, BAF53a, and Oct4 after 4-hydroxytamoxifen (OHT)-mediated Cre recombinase induction. Click here for file

Additional file 5 **Replication domains that are commonly dysregulated both in *BAF250a *** **and *Brg1 *** **mutants.** Venn diagrams show the overlap between domains that undergoes early to late (EtoL) switching (left) and late to early (LtoE) switching (right) upon BAF250a loss in embryonic stem cells (ESCs) versus Oct4, Sox2, Klf4, and c-Myc (OSKM) cells (false discovery rate (FDR) = 1% from Figure 1C and Figure 2F), as compared to Brg1-affected EtoL and LtoE segments (FDR = 1% from Figure 3D). Since the total number of affected segments is small, the overlap between BAF250a-affected EtoL segments and Brg1-affected EtoL segments is highly significant relative to what would be expected for a random distribution (*P* <0.001). However, limited overlaps between these segments may also suggest the existence of subunit-specific roles in replication timing regulation. Click here for file

Additional file 6 **Replication timing of the Oct4 domain.** Replication timing profile of the *Oct4* domain derived from genome-wide analysis of various cell types. The domain is early replicating before and after esBAF complex deficiency. Thirty seven Brg1 binding sites were identified by chromatin immunoprecipitation (ChIP)-seq [35] within the domain. Click here for file
