# Supplementary figures and images for: Murine esBAF chromatin remodeling complex subunits BAF250a and Brg1 are necessary to maintain and reprogram pluripotency-specific replication timing of select replication domains
Source: Epigenetics Chromatin. 2013 Dec 13;6:42. doi: 10.1186/1756-8935-6-42 (PMC3895691; doi:10.1186/1756-8935-6-42)

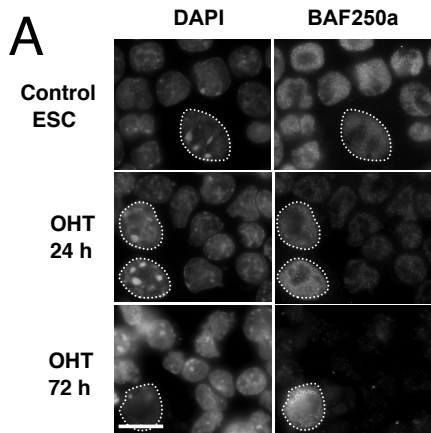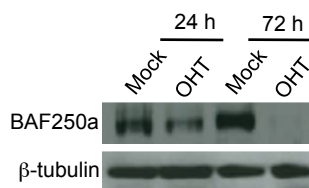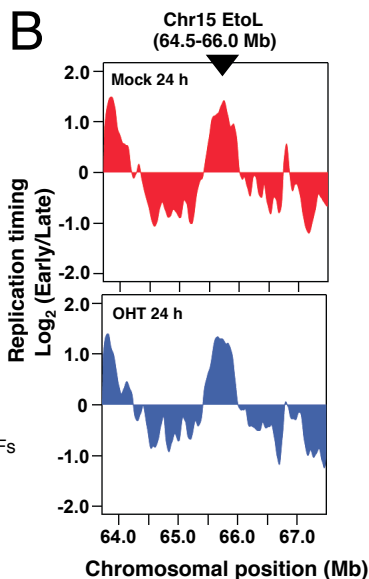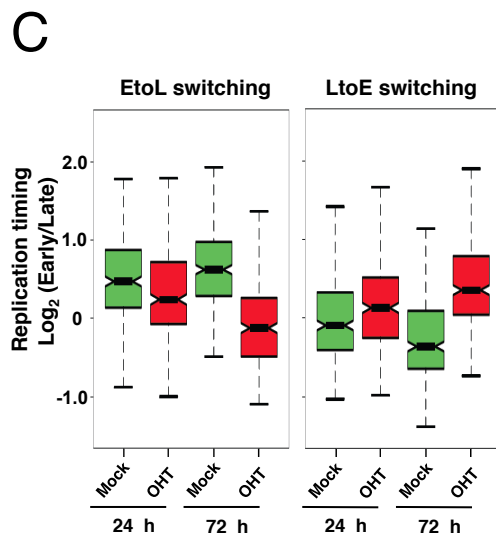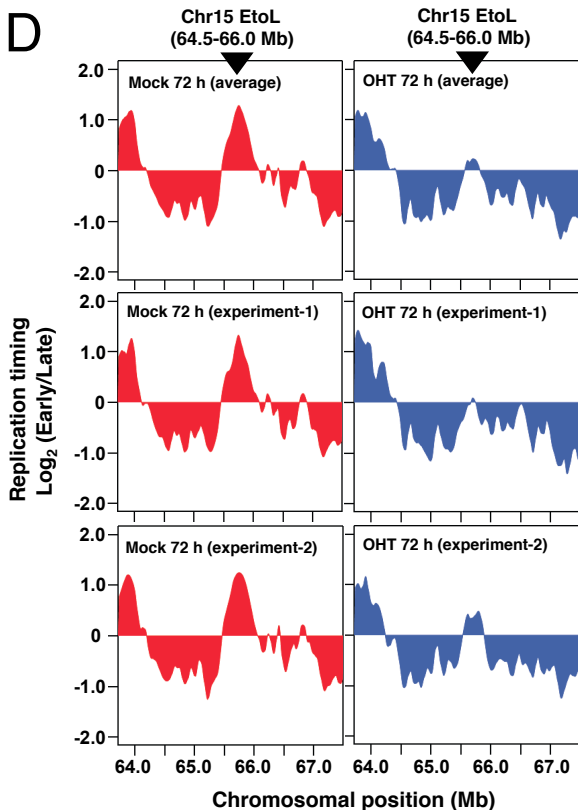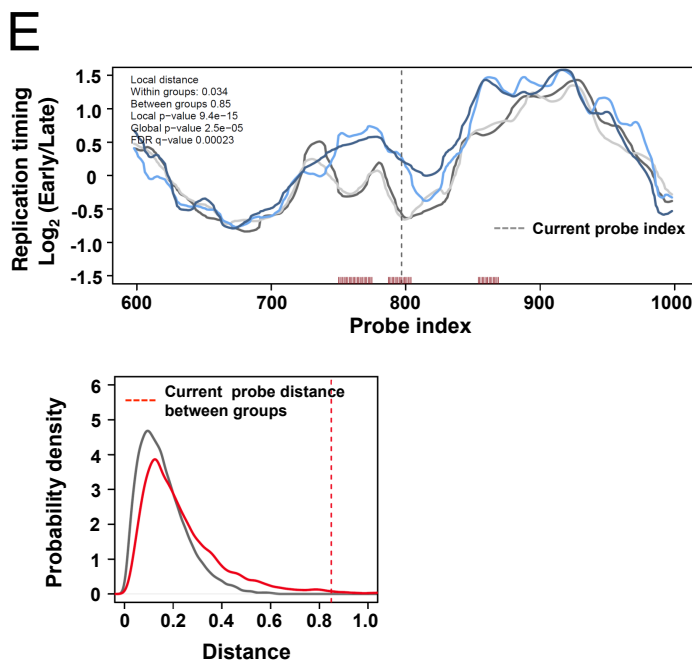

Supplement: Additional file 1 — Replication timing profile at chr15 (65.4-66.0 Mb) domain. (A) TOP: The BAF250a protein level was monitored by immunofluorescence staining at 0 (control), 24, and 72 h after 4-hydroxytamoxifen (OHT)-mediated induction of Cre recombinase. Circled are the nuclei of feeder mouse embryonic fibroblasts (MEFs), in which BAF250a protein level is not affected by the drug treatment, which serves as an internal immunostaining control. Bars, 10 μm. BOTTOM: Western blot showing protein levels of BAF250a with (OHT) and without (Mock) Cre recombinase induction. (B) Replication timing profile of the chr15 domain shown in Figure 1D from untreated, 24 h mock-treated and 24 h OHT-treated embryonic stem cells (ESCs). Replication timing change at this domain was not observed during the 24 h experimental period. (C) Box plots show the replication timing of domains that are sensitive to BAF250a loss (false discovery rate (FDR) = 1% from Figure 1C) after 24 h and 72 h of OHT treatment. (D) Responses to BAF250a loss are reproducible. Top panels show average replication timing profile at the Chr15 domain. Replication timing profiles from two independent experiments are shown below. (E)P value calculation based on the global Euclidian distances between groups and within replicates. Top plot is an examplary region showing replication timing of BAF250 ESC OHT in dark and light blue, and BAF250 ESC mock in dark and light grey. Bottom plot shows global Probability Density Function (PDF) of Euclidian distance between groups (red) and within replicates (grey) calculated from replication timing in individual probes. The individual probes with significant replication timing differences are shown as red lines in the top plot. [file 1756-8935-6-42-S1.pdf]

**Chr16 *Dppa2/4* domain**

**Chr8 *Rex1* domain**

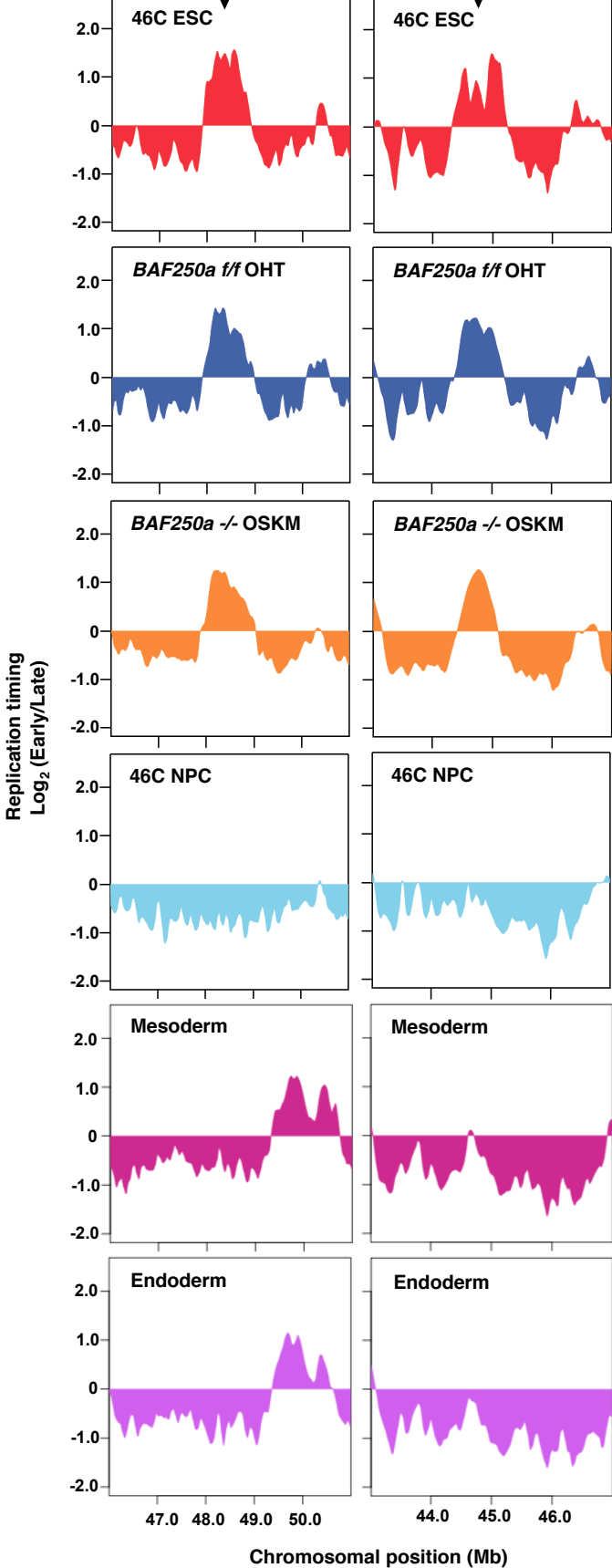

Supplement: Additional file 2 — BAF250a -deficient embryonic stem cells (ESCs) have pluripotency-specific replication profiles. Replication timing profile of Dppa2/4 and Rex1 domains derived from genome-wide analysis of various cell types. These domains are known to show early replication in pluripotent cells, but switch to late replication after differentiation [6]. [file 1756-8935-6-42-S2.pdf]

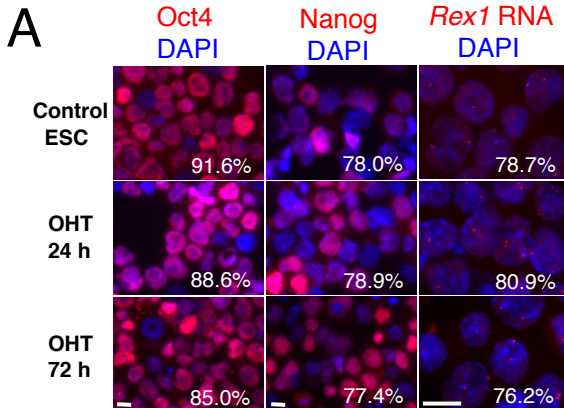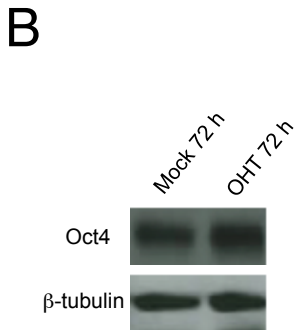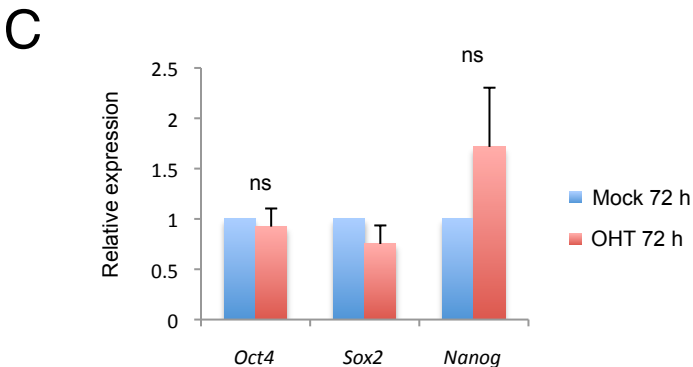

Supplement: Additional file 3 — Pluripotency-associated marker expressions in BAF250a -deficient embryonic stem cells (ESCs). (A) Immunofluorescence analysis of Oct4 and Nanog proteins (left two panels) and RNA-FISH analysis of Rex1 mRNA in mock- and 4-hydroxytamoxifen (OHT)-treated ESCs. Bars, 10 μm. (B) Western blot showing protein levels of Oct4. The results for the loading control, tubulin, were the same as those in Additional file 1A. (C) RT-PCR expression level validation for pluripotency-associated genes. *P <0.05; nsP >0.05 (no significant difference). Statistical analysis was performed by a two-tailed Student’s t-test. [file 1756-8935-6-42-S3.pdf]

*Brg1*  
ESC

Mock OHT

Brg1

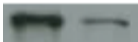

Oct4

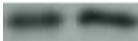

$\beta$ -tubulin

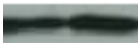

*BAF53a*  
ESC

Mock OHT

BAF53a

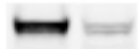

Oct4

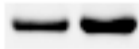

Histone H3

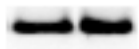

Supplement: Additional file 4 — Characterization of conditional Brg1 and BAF53a knockout. Western blot showing protein levels of Brg1, BAF53a, and Oct4 after 4-hydroxytamoxifen (OHT)-mediated Cre recombinase induction. [file 1756-8935-6-42-S4.pdf]

Chr17 *Oct4* domain

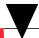

Replication timing  
 $\text{Log}_2$  (Early/Late)

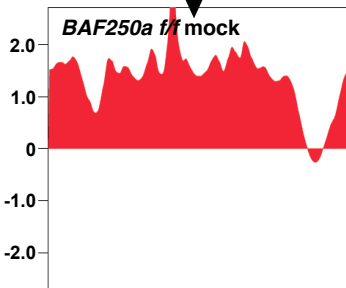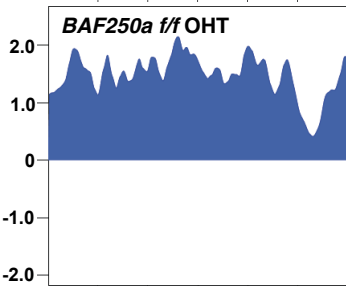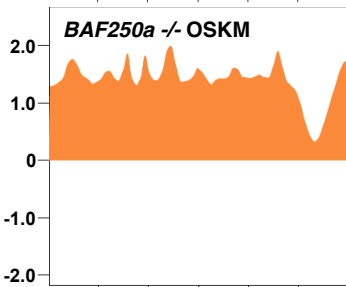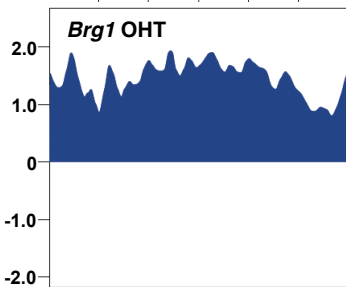

34.0 34.5 35.0 35.5 36.0

Chromosomal position (Mb)

Supplement: Additional file 6 — Replication timing of the Oct4 domain. Replication timing profile of the Oct4 domain derived from genome-wide analysis of various cell types. The domain is early replicating before and after esBAF complex deficiency. Thirty seven Brg1 binding sites were identified by chromatin immunoprecipitation (ChIP)-seq [35] within the domain. [file 1756-8935-6-42-S6.pdf]
