# Supplementary material for: Murine esBAF chromatin remodeling complex subunits BAF250a and Brg1 are necessary to maintain and reprogram pluripotency-specific replication timing of select replication domains
Source: Epigenetics Chromatin. 2013 Dec 13;6:42. doi: 10.1186/1756-8935-6-42 (PMC3895691; doi:10.1186/1756-8935-6-42)
Supplement: Additional file 5 — Replication domains that are commonly dysregulated both in BAF250a and Brg1 mutants. Venn diagrams show the overlap between domains that undergoes early to late (EtoL) switching (left) and late to early (LtoE) switching (right) upon BAF250a loss in embryonic stem cells (ESCs) versus Oct4, Sox2, Klf4, and c-Myc (OSKM) cells (false discovery rate (FDR) = 1% from Figure 1C and Figure 2F), as compared to Brg1-affected EtoL and LtoE segments (FDR = 1% from Figure 3D). Since the total number of affected segments is small, the overlap between BAF250a-affected EtoL segments and Brg1-affected EtoL segments is highly significant relative to what would be expected for a random distribution (P <0.001). However, limited overlaps between these segments may also suggest the existence of subunit-specific roles in replication timing regulation. [file 1756-8935-6-42-S5.pdf]

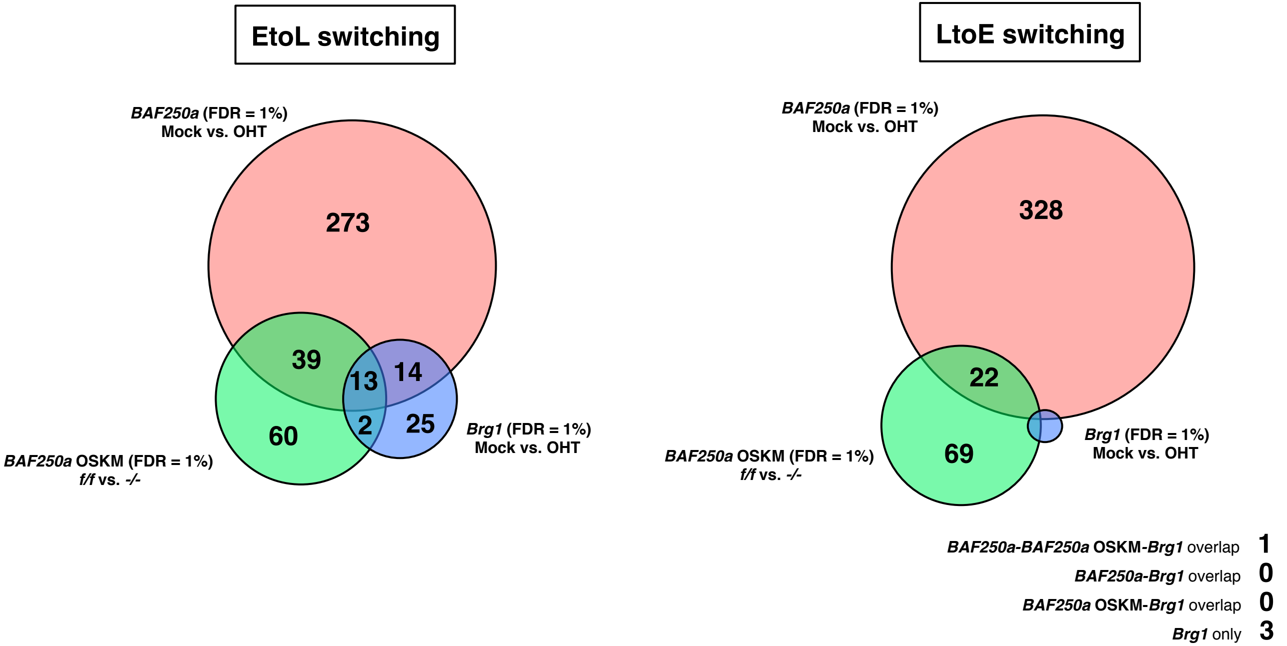
EtoL switching
BAF250a (FDR = 1%)
Mock vs. OHT
273
39
13
14
60
2
25
Brg1 (FDR = 1%)
Mock vs. OHT
BAF250a OSKM (FDR = 1%)
f/f vs. *-/-*
LtoE switching
BAF250a (FDR = 1%)
Mock vs. OHT
328
22
69
Brg1 (FDR = 1%)
Mock vs. OHT
BAF250a OSKM (FDR = 1%)
f/f vs. *-/-*
BAF250a-*BAF250a* OSKM-*Brg1* overlap 1
BAF250a-*Brg1* overlap 0
BAF250a OSKM-*Brg1* overlap 0
Brg1 only 3
